# Dose-Dependent Efficacy of Nefopam for Preventing Catheter-Related Bladder Discomfort in Patients Undergoing Transurethral Ureteroscopic Lithotripsy: A Retrospective Case–Control Observational Study

**DOI:** 10.3390/jcm15083099

**Published:** 2026-04-18

**Authors:** Jae Hun Hwang, Hyung Rae Cho, Ju-Yeun Lee, Seo Yeon Lee, Jiyoung Kim

**Affiliations:** 1 Department of Anesthesiology and Pain Medicine, Myongji Hospital, College of Medicine, Hanyang University, Goyang 10475, Republic of Korea; rjqnrdl4248@naver.com (J.H.H.); callmex@hanmail.net (H.R.C.); 2 Department of Ophthalmology, Myongji Hospital, College of Medicine, Hanyang University, Goyang 10475, Republic of Korea; leejy5293@gmail.com; 3 Department of Preventive Medicine, College of Medicine, Seoul National University, Seoul 03080, Republic of Korea; 4 Integrated Major in Innovative Medical Science, College of Medicine, Seoul National University, Seoul 03080, Republic of Korea; 5 Department of Urology, Myongji Hospital, College of Medicine, Hanyang University, Goyang 10475, Republic of Korea; photomol@hanmail.net

**Keywords:** dose–response relationship, drug, litholapaxy, nefopam, ureteroscopy, urinary catheterization

## Abstract

**Background/Objectives**: Catheter-related bladder discomfort (CRBD) is a common complication that patients with Foley catheters may experience following surgery. Previous studies have suggested that nefopam can reduce the incidence and severity of CRBD; however, dose-dependent effects (20 mg vs. 40 mg) have not been directly compared. Therefore, this study aimed to evaluate the dose-dependent effects of nefopam on CRBD, determine its effective dose, and assess the incidence of associated side effects. **Methods**: Electronic medical records of patients aged 18–70 years with American Society of Anesthesiologists physical status I–III who underwent elective transurethral ureteroscopic lithotripsy under general anesthesia from August 2016 to December 2022 were reviewed. Patients were categorized into three groups: premedication with intravenous nefopam 20 mg (group N20), premedication with nefopam 40 mg (group N40), or no premedication (control, group C). **Results**: The incidence rates of CRBD were 85.7% in group C, 81.3% in group N20, and 51.4% in group N40, showing a significant difference among the groups (*p* = 0.003, Pearson’s chi-squared test). Postoperative NRS was significantly different among the groups (*p* < 0.001, one-way ANOVA). In post hoc analysis, both group N20 and group N40 showed significantly lower scores compared to group C (*p* = 0.002, *p* = 0.001 respectively). The severity of CRBD also decreased in a dose-dependent manner, which was considered significant. No significant differences were observed among the groups in terms of intraoperative hemodynamic stability or postoperative nausea and vomiting. **Conclusions**: The administration of nefopam 40 mg significantly reduced the incidence and severity of CRBD compared with no premedication.

## 1. Introduction

Catheter-related bladder discomfort (CRBD) can exacerbate postoperative pain and delay recovery in patients undergoing transurethral ureteroscopic lithotripsy. CRBD is characterized by a burning sensation in the suprapubic region and an urge to void [1]. It is caused by involuntary bladder spasms mediated through muscarinic receptor activity in the bladder muscles surrounding the catheter [2]. As a postoperative complication, CRBD can cause emergency agitation in the post-anesthesia care unit (PACU), significantly increasing the workload of PACU staff, and therefore requires aggressive management [3].

Nefopam is a centrally acting, non-opioid, non-steroidal antinociceptive drug [4]. Its mechanism of action resembles that of triple reuptake inhibitors (affecting serotonin, norepinephrine, and dopamine pathways) and shares similarities with certain anticonvulsants. Previous studies, including ours, have demonstrated the prophylactic effect of nefopam against CRBD [5,6]. However, limitations remain, as dose-dependent effects (20 mg vs. 40 mg) and the incidence of side effects have not been directly compared. Given the significant variability in body weight among patients undergoing lithotripsy and bladder catheterization, the current fixed dosing of nefopam (20 mg or 40 mg) remains dichotomous, necessitating a weight-based guideline of nefopam for preventing CRBD.

Therefore, this retrospective observational study aimed to investigate the effect of nefopam dose on CRBD and assess the associated incidence of side effects. Further, we sought to investigate a weight-based guideline for dosing nefopam.

## 2. Materials and Methods

### 2.1. Study Design

The study was reviewed and approved by the Institutional Review Board of Myongji Hospital (approval number: MJH2023-03-031). All data were retrospectively obtained from electronic medical records, and patients were not publicly enrolled prior to data collection. Written informed consent was waived owing to the retrospective nature of the study. Electronic medical records of patients who underwent transurethral ureteroscopic lithotripsy were reviewed. All patients who met the eligibility criteria were enrolled in the study. Patients aged 18–70 years with American Society of Anesthesiologists physical status I–III who underwent elective transurethral ureteroscopic lithotripsy under general anesthesia between August 2016 and December 2022 were analyzed. Patients were categorized into the control group (no nefopam), N20 group (nefopam 20 mg), and N40 group (nefopam 40 mg) according to the preoperative nefopam administration status and dose administered. Conversely, patients with a history of chronic pain, drug or alcohol abuse, psychiatric conditions, morbid obesity (body mass index > 30 kg/m^2^), epilepsy, angle-closure glaucoma, heart failure, arrhythmia, myocardial infarction, bladder outflow obstruction, overactive bladder (OAB) (frequency > 3 times per night or >8 times per 24 h), renal and hepatic diseases, and current use of monoamine oxidase inhibitors were excluded. In order to ensure data integrity of this study, we retrospectively assessed whether there were any deviations from our institutional anesthetic protocol. Any cases that significantly strayed from our institutional protocol were excluded from the analysis.

### 2.2. Anesthesia Protocol

Anesthesia was administered by the anesthesiologist assigned on the day of surgery, following the department’s routine protocol, with the exception of nefopam premedication. Upon arrival in the operating room, patients received 40 mg or 20 mg of nefopam, diluted in 100 mL of 0.9% saline, administered intravenously at a rate of 150 mL/h. Administration of nefopam dose (0 mg, 20 mg, 40 mg) was determined by anesthesiologists’ clinical judgement in accordance with institutional clinical protocol. These decisions were guided by individual patient characteristics and established evidence regarding the dose-dependent analgesic effects of nefopam [5,6]. To reduce pain during nefopam injection, the solution was warmed and infused using two fluid warmers, as described by Cheon et al. [6]. No other medications were administered prior to induction of anesthesia. Anesthesia was induced with 2 mg/kg propofol and 0.8 mg/kg rocuronium bromide, followed by insertion of a laryngeal mask airway (LMA, I-gel^TM^, Intersurgical Ltd., Wokingham, UK). Anesthesia was maintained with 2.0–3.0 vol% sevoflurane, targeting a bispectral index of 40–60. The fraction of inspired oxygen was set to 0.5. Intraoperative bradycardia (heart rate < 40 beats/min) was treated with 0.25 or 0.5 mg atropine, whereas hypotension (mean arterial pressure < 65 mmHg) was treated with 5 to 10 mg ephedrine. Hypertension, defined as systolic blood pressure > 140 mmHg or diastolic blood pressure > 90 mmHg, was managed with nicardipine or labetalol as appropriate. Tachycardia (heart rate > 100 beats/min) was treated by increasing the inhaled anesthetic concentration and administering an additional 10 mg of esmolol as needed. Following ureteroscopic removal of ureteral stones, a 6 Fr ureteral stent and a 16 Fr urethral catheter were inserted in all patients. Muscle relaxation was reversed with 2 mg/kg sugammadex. After confirmation of spontaneous respiration and an eye opening, the LMA was removed, and patients were transferred to the PACU.

### 2.3. Outcomes

The primary endpoint was the incidence of CRBD, whereas the secondary endpoints were the numeric rating scale (NRS) score for postoperative pain, the severity of CRBD, pethidine rescue dose, and side effects. The incidence and severity of CRBD and the NRS score for postoperative pain were assigned by the PACU nurse blinded to group allocation at 10 min intervals for 1 h, provided the patient’s sedation level was 1 or 2 according to the Ramsay sedation score. The highest values recorded during the observation period were used for analysis. Postoperative pain was measured using the NRS (0 = no pain; 10 = worst imaginable pain). CRBD severity was classified as follows: none, if no complaints were reported; mild, if CRBD was reported only when specifically asked; moderate, if CRBD was self-reported; and severe, if CRBD was reported and accompanied by attempts to remove the catheter, involuntary movements of the hands and feet, or vocal expressions of pain. Patients with an NRS score of ≥5 received repeated intravenous injections of 25 mg pethidine until the score reduced below 5. Intraoperative hypertension, tachycardia, postoperative headache, nausea and vomiting (PONV), somnolence, blurred vision, and hyperhidrosis were also recorded.

### 2.4. Statistical Analyses

Sample size was determined using G*Power (version 3.1.9.4). To detect a medium effect size (w = 0.3) for the incidence of CRBD with an α error of 0.05 and a power (1 − β) of 0.80, a total sample size of 108 patients was recommended. In this study, 102 patients (35 in group C, 32 in group N20, and 35 in group N40) were ultimately enrolled and analyzed. This sample size was considered sufficient to detect clinically meaningful differences among the groups.

Statistical analyses were performed using SPSS Statistics software (version 28.0 for Windows, IBM Corp., Armonk, NY, USA). Demographic characteristics, duration of surgery and anesthesia, rescue pethidine dose, and pain NRS scores were compared among the three groups using a one-way analysis of variance. Post hoc comparisons were performed using the Scheffé or Dunnett’s T3 test, as appropriate. When the assumption of normality was not satisfied and a non-parametric test was required, the Kruskal–Wallis test was used. Categorical variables were analyzed using the χ^2^ test, the χ^2^ test for trends (linear-by-linear association), or Fisher’s exact test, as appropriate. Probit regression analysis was performed to determine the effective dose of nefopam. A *p* value of <0.05 was considered significant.

## 3. Results

**Figure 1:**
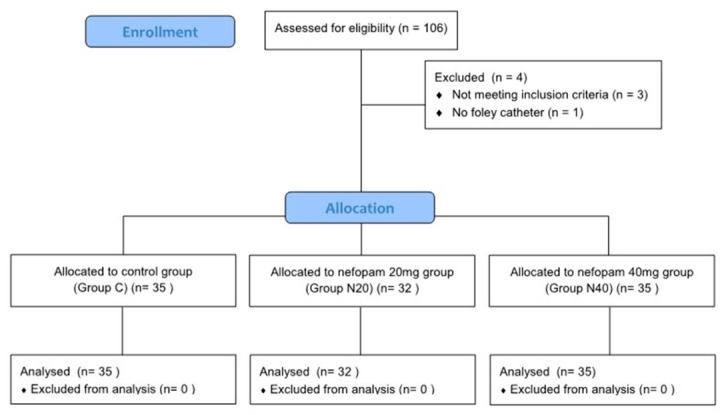
Flowchart illustrating the patient selection process.

**Figure 2:**
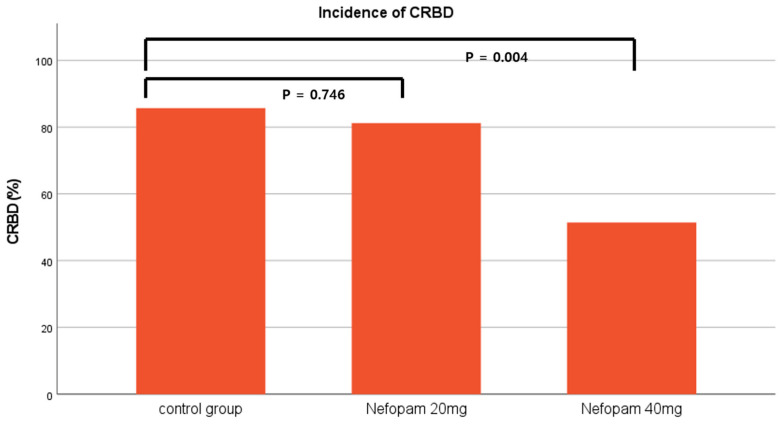
Incidence of catheter-related bladder discomfort among the three groups.

**Figure 3:**
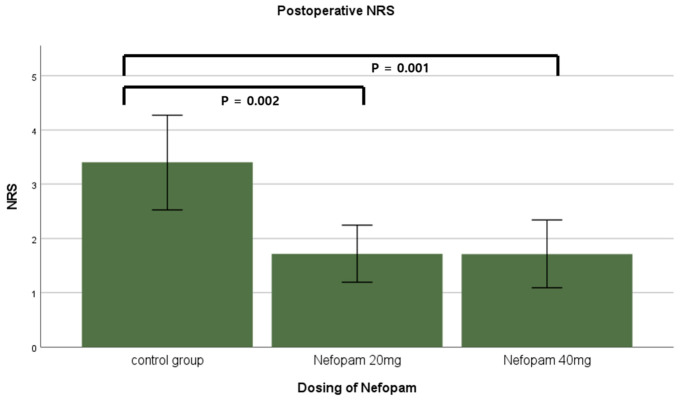
Comparison of postoperative numeric rating scale scores among the three groups. Error bars indicate 95% confidence intervals. NRS: numeric rating scale.

**Figure 4:**
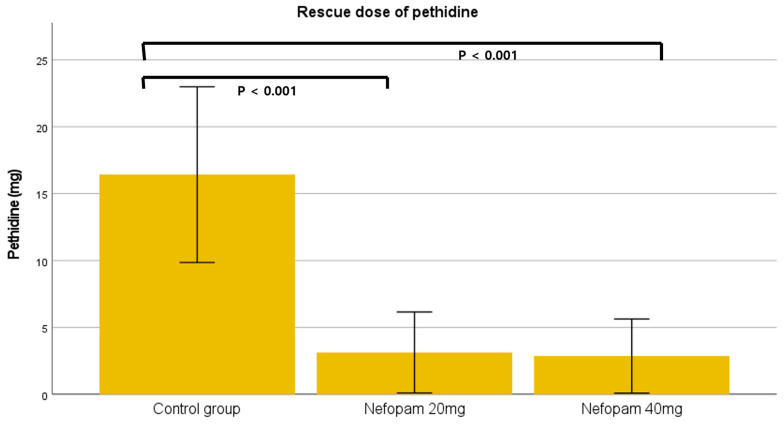
Comparison of pethidine rescue doses among the three groups. Error bars indicates 95% confidence intervals.

**Figure 5:**
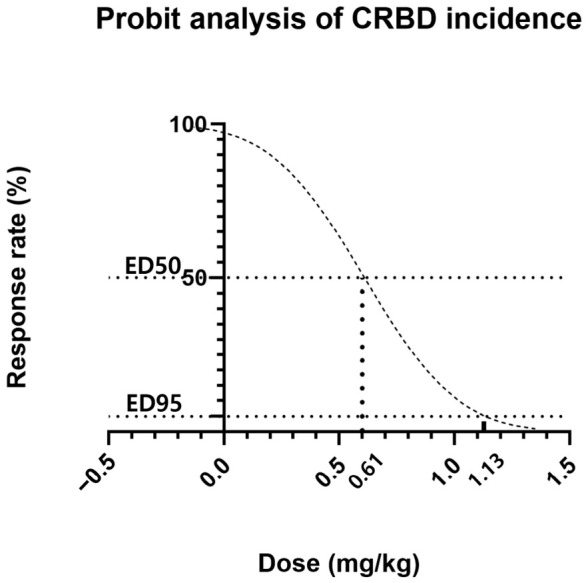
Probit analysis of ED50 and ED95 of nefopam for CRBD prevention based on patients’ weight. The sigmoid curve represents the predicted probabilities of incidence of CRBD in relation to nefopam dose. As the dose of nefopam increases, the probability of CRBD incidence decreases. ED50 (0.61 mg/kg) and ED95 (1.13 mg/kg) are indicated in dotted lines, representing the doses associated with a 50% and 5% predicted incidence of CRBD, respectively. CRBD: catheter-related bladder discomfort, ED: effective dose.

A total of 106 patients who underwent ureteroscopic litholapaxy under general anesthesia during the study period were initially identified by screening the electronic medical records. Four patients were excluded based on the exclusion criteria, resulting in a final cohort of 102 patients for final analysis. A total of 35 patients were assigned to the control group (group C), 32 to the nefopam 20 mg group (group N20), and 35 to the nefopam 40 mg group (group N40) (Figure 1). No significant difference was observed in the demographic characteristics among the three groups (Table 1). The incidence rates of CRBD were 85.7% in group C, 81.3% in group N20, and 51.4% in group N40, with a significant difference noted between the groups (*p* = 0.003). A dose-dependent decrease in CRBD incidence was observed as the nefopam dose increased, which was significant (*p* = 0.002, Figure 2) (Table 2). Similarly, severity demonstrated a significant decreasing trend with increasing nefopam dose in the trend test (*p* < 0.001). The maximum postoperative NRS scores were 3.4 ± 2.5 in group C, 1.7 ± 1.5 in group N20, and 1.6 ± 1.8 in group N40. Post hoc analysis revealed that group C differed significantly from both group N20 and group N40 (*p* < 0.001) (Figure 3, Table 2). The mean pethidine rescue doses required for postoperative pain were 16.5 ± 18.1 mg in group C, 2.8 ± 7.7 mg in group N20, and 2.8 ± 8.1 mg in group N40, with post hoc comparisons again showing significant differences between group C and group N20 or group N40 (*p* < 0.001, Figure 4) (Table 2). Assessment of intraoperative hemodynamic stability, including hypertension, hypotension, and tachycardia, revealed no significant differences among the groups. Similarly, the incidence of postoperative nausea and vomiting (PONV) did not differ significantly between the three groups (Table 3). The effective dose (ED) of nefopam preventing CRBD was calculated using probit analysis (Figure 5). ED50 and ED95 values were approximately 0.6 mg/kg and 1.1 mg/kg, respectively.

## 4. Discussion

This present study found that the administration of nefopam 40 mg significantly reduced the incidence of CRBD compared with the control group, whereas nefopam 20 mg did not demonstrate a significant effect. As the nefopam dose increased, the severity of CRBD and the frequency of pethidine rescue analgesia decreased. No significant differences were observed in the incidence of adverse effects among the three groups.

CRBD is known to result from involuntary bladder contractions triggered by catheter-induced bladder irritation, primarily mediated by muscarinic receptor activation. Its pathophysiology closely resembles that of an OAB in terms of urotheliogenic mechanisms. In OAB, acetylcholine released from activated cholinergic nerves stimulates detrusor muscle contraction via muscarinic receptors located within the urothelium/suburothelium [2]. The urothelium functions as a sensory structure capable of detecting mechanical, chemical, and thermal stimuli [7]. CRBD is a common postoperative complication that may cause agitation and pain in the PACU; therefore, active management is warranted [3].

**Figure 6:**
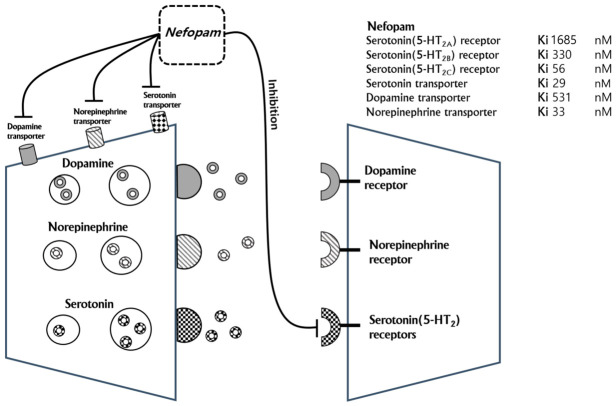
Proposed mechanism of action of nefopam. Ki: inhibition constant; a smaller Ki indicates a higher binding affinity, requiring a lower drug concentration to inhibit the enzyme’s activity.

Numerous studies have investigated various strategies for managing CRBD, including non-pharmacological and pharmacological methods. Non-pharmacological interventions include the use of novel urinary catheters and the selection of smaller urinary catheters [8,9]. Several medications have been reported to be effective in preventing CRBD, and meta-analyses have demonstrated significant efficacy for agents such as antimuscarinics, gabapentin, tolterodine, ketamine, trospium, dexmedetomidine, oxybutynin, and nefopam [5,6,10,11,12,13,14,15,16,17,18]. Among these, nefopam is a well-established agent used in the field of anesthesia to prevent emergence agitation and shivering following spinal anesthesia or to reduce opioid requirements for intraoperative and postoperative analgesia [19,20,21,22,23,24]. The pharmacological mechanism of nefopam resembles that of the triple reuptake receptor (serotonin, norepinephrine, and dopamine) and certain anticonvulsants [25] (Figure 6). A study investigating the primary targets of nefopam reported its inhibitory affinity of nefopam in the following order: serotonin transporter > norepinephrine transporter > serotonin (5-HT)2C receptor > 5-HT2B receptor > dopamine transporter > 5-HT2A receptor [26]. Specifically, the inhibition of the 5-HT2C receptor enhances the release of dopamine and norepinephrine. Therefore, the CRBD-preventive effect of nefopam was achieved through multiple mechanisms. First, nefopam inhibits serotonin reuptake transporters and serotonin receptors—particularly serotonin receptors 2C, 2B, and 2A—thereby increasing serotonin levels in the central nervous system and suppressing bladder activity. Second, elevated norepinephrine levels stimulate beta-3 adrenergic receptors, resulting in relaxation of the detrusor muscle [27]. Third, inhibition of dopamine reuptake increases the concentration of dopamine in the synaptic cleft, which further contributes to detrusor muscle relaxation.

Previous studies have investigated the efficacy of 20 mg of nefopam; however, this dose was insufficient to reduce the incidence of CRBD. A study involving patients who underwent robotic nephrectomy failed to draw definitive conclusions regarding the effect of nefopam on CRBD incidence [28]. As shown in our study, the 20 mg dose did not reduce the incidence of CRBD compared with the control group but only alleviated its severity. Although several studies have demonstrated the efficacy of nefopam 20 mg [5], the potential influence of spinal anesthesia could not be completely excluded [9]. In a dose–response study involving patients undergoing moderately painful surgery, the ED80 was approximately 60 mg, which is considerably higher than the commonly used 20 mg. Similarly, a study on laparoscopic cholecystectomy reported an ED50 of approximately 60 mg [29,30]. Therefore, the effective dose of nefopam for preventing CRBD was determined based on the findings of this study. After converting the administered nefopam dose to mg/kg of body weight, the ED50 and ED95 values were derived using probit regression (Supplementary Table S1). The calculated ED50 and ED95 values were approximately 0.6 mg/kg and 1.1 mg/kg, respectively. Therefore, a nefopam dose of 1.1 mg/kg is recommended to prevent the occurrence of CRBD in 95% of patients who underwent ureteroscopic litholapaxy under general anesthesia.

This study has some limitations. First, owing to the limitations of retrospective studies, the Foley catheter size and use of ureteral stents could not be standardized. Second, the outcomes were assessed only in the PACU, and delayed CRBD was not evaluated. Third, although the anesthesia and surgical methods were similar, it was not possible to completely control for variations caused by the anesthesiologist or surgeon. Fourth, the retrospective nature of the study may have resulted in a relatively small sample size, reducing the statistical power of the findings. Post hoc analysis indicated that the power for comparisons between the control and N40 groups, as well as between the N20 and N40 groups, exceeded 0.99; however, the power for the control vs. N20 comparison was <0.3. Therefore, a larger sample size is needed to more accurately assess the efficacy of nefopam 20 mg in preventing CRBD. Additionally, the incidence of nefopam-related side effects was relatively low and was not subjected to statistical analysis for comparison between the control, N20, and N40 groups. Therefore, further comprehensive studies are warranted to evaluate and compare the safety profile between the N20 and N40 groups.

## 5. Conclusions

In conclusion, the administration of 40 mg of nefopam significantly reduced both the incidence and severity of CRBD compared with the control group. To prevent CRBD in 95% of patients under general anesthesia, a nefopam dose of 1.1 mg/kg is recommended.

## Figures and Tables

**Table 1:** Demographic data.

Parameters	Control (*n* = 35)	N20 (*n* = 32)	N40 (*n* = 35)	*p* Value
Age (Yr)	48.4 ± 14.9	55.4 ± 14.8	52.8 ± 11.3	0.115
Sex (M/F)	25/10	17/15	19/16	0.264
ASA classification (I/II/III)	19/16/0	19/10/3	18/17/0	0.142
Weight (kg)	71.3 ± 10.2	67.4 ± 13.7	67.6 ± 10.5	0.296
Height (cm)	168.7 ± 7.6	163.9 ± 9.5	162.8 ± 9.0	0.014
BMI (kg/m^2^)	24.6 ± 2.7	24.9 ± 4.0	25.4 ± 2.8	0.578
Stone size (mm)	5.7 ± 1.7	6.2 ± 2.9	5.6 ± 1.4	0.583
Ureter stone position				0.303
Upper	8	10	5	
Mid	2	3	6	
Lower	25	18	24	
Duration of operation (min)	28.3 ± 22.7	23.5 ± 17.6	22.1 ± 16.4	0.761
Duration of anesthesia (min)	36.4 ± 25.5	35.3 ± 18.2	39.2 ± 16.1	0.264

Data are presented as mean ± SD values or the number of patients. BMI: body mass index, ASA: American Society of Anesthesiology.

**Table 2:** Incidence and severity of catheter-related bladder discomfort.

	Control (*n* = 35)	N20 (*n* = 32)	N40 (*n* = 35)	OR (95% CI)			*p* Value
				Control vs. N20	Control vs. N40	N20 vs. N40	
CRBD							
Incidence	30 (85.7)	26 (81.3)	18 (51.4)	0.722 (0.197–2.644)	0.176 (0.056–0.561)	0.244 (0.081–0.740)	0.003
Severity							<0.001
Mild	11 (31.4)	19 (59.4)	15 (42.9)				
*Moderate*	*15 (42.9)*	*7 (21.9)*	*3 (8.6)*				
Severe	4 (11.4)	0 (0)	0 (0)				
Postoperative NRS	3.4 ± 2.5	1.7 ± 1.5	1.6 ± 1.8				<0.001
Rescue pethidine dose (mg)	16.5 ± 18.1	2.8 ± 7.6	2.8 ± 8.1				<0.001

Data are presented as mean ± SD values or the number of patients (%). CRBD: catheter-related bladder discomfort, NRS: numerical rating scale, e, CI: confidence interval, OR: odds ratio.

**Table 3:** Incidence of side effects.

Parameters	Control (*n* = 35)	N20 (*n* = 32)	N40 (*n* = 35)	*p* Value
Intraoperative				
Hypotension	2 (5.7)	0 (0)	0 (0)	0.327
Hypertension	17 (48.6)	22 (68.8)	23 (65.7)	0.185
Tachycardia	4 (11.4)	10 (31.3)	4 (11.4)	0.074
Postoperative				
Nausea and vomiting	1 (2.9)	0 (0.0)	0 (0.0)	1.000
Headache	0 (0.0)	0 (0.0)	0 (0.0)	N/A
Somnolence	0 (0.0)	0 (0.0)	0 (0.0)	N/A
Blurred vision	0 (0.0)	0 (0.0)	0 (0.0)	N/A
Hyperhidrosis	0 (0.0)	0 (0.0)	0 (0.0)	N/A

Data are presented as the number of patients (%). Hypertension: systolic blood pressure > 140 mmHg or diastolic blood pressure > 90 mmHg. Tachycardia: heart rate > 100 beats/min. N/A: not applicable.

## Data Availability

The data presented in this study are available on request from the corresponding author due to privacy concerns.

## Supplementary Materials

The following supporting information can be downloaded at: https://zenodo.org/records/18604406, Table S1: Results of predicting effective dose ED50 and ED95 of nefopam for CRBD prevention using probit analysis.

## Abbreviations

The following abbreviations are used in this manuscript:

ASA	American Society of Anesthesiologists
CRBD	Catheter-related bladder discomfort
ED	Effective dose
ED50	Effective dose for 50% of the population
ED80	Effective dose for 80% of the population
ED95	Effective dose for 95% of the population
FR	French (catheter size unit)
LMA	Laryngeal mask airway
NRS	Numeric rating scale
OAB	Overactive bladder
PACU	Post-anesthesia care unit
PONV	Postoperative nausea and vomiting
SPSS	Statistical Package for the Social Sciences
5-HT	5-hydroxytryptamine
